# Idiopathic Subglottic Stenosis During Pregnancy: Successful Treatment With Green Laser Ablation and Steroid Injection

**DOI:** 10.7759/cureus.79788

**Published:** 2025-02-27

**Authors:** Makoto Miyamoto, Atsushi Tajima, Tomoki Naoi, Misa Koshihara, Akira Motoyasu

**Affiliations:** 1 Otorhinolaryngology, ISEIKAI International General Hospital, Osaka, JPN; 2 Obstetrics and Gynecology, Kyorin University School of Medicine, Tokyo, JPN; 3 Otolaryngology - Head and Neck Surgery, Kyorin University School of Medicine, Tokyo, JPN; 4 Anesthesiology, Kyorin University School of Medicine, Tokyo, JPN

**Keywords:** endoscopic treatment using laser, idiopathic subglottic stenosis, multidisciplinary medical team, #pregnant women, treatment strategies

## Abstract

Idiopathic subglottic stenosis (ISGS) during pregnancy is extremely rare, with treatment strategies primarily based on a limited number of case reports. The most common management approach in pregnant women is therapeutic endoscopic treatment with a balloon catheter. This intervention is crucial for the well-being of both the mother and fetus and requires a multidisciplinary team, including specialists in obstetrics, anesthesiology, and otolaryngology. We present the case of a 31-year-old woman with ISGS at 28 weeks of pregnancy. Throughout the procedure, obstetricians performed fetal monitoring, while anesthesiologists managed her respiration and pain. The patient was successfully treated with laser ablation and steroid injection using flexible endoscopy under local anesthesia. She subsequently had a spontaneous vaginal delivery at 38 weeks and three days, giving birth to a healthy boy. This case highlights the successful management of symptomatic subglottic stenosis during pregnancy through endoscopic laser ablation and localized steroid injection.

## Introduction

Subglottic stenosis (SGS) typically occurs as a secondary complication of tracheal intubation, tracheostomy, or polyangiitis (Wegener granulomatosis) [1,2]. It is characterized by circumferential cicatricial stenosis of the subglottic larynx and proximal trachea [3]. Idiopathic SGS (ISGS) is a diagnosis of exclusion with an unknown cause [4] and is an extremely rare condition. It predominantly affects women [5,6], with most reported cases in pregnant women appearing as isolated case reports.

In this case, a 31-year-old woman experienced worsening symptoms, including breathing difficulties and wheezing, during her second pregnancy. Endoscopic laser surgery and local steroid injections were performed in the third trimester to manage SGS, and she subsequently had a spontaneous vaginal delivery. This report details the clinical presentation, radiographic findings, endoscopic laser treatment, and the role of endoscopy in managing ISGS during pregnancy.

## Case presentation

Patient history

A 31-year-old woman became pregnant with her first child three years ago and gave birth at Obstetrics Clinic A. During pregnancy, she experienced respiratory symptoms, including stridor and difficulty breathing, and was treated for asthma. After childbirth, she was diagnosed with SGS through flexible endoscopy and histological examination at the Department of Thoracic Surgery at B University Hospital. However, her respiratory symptoms persisted, and she was monitored without medication or surgical intervention.

During her second pregnancy, she developed pneumonitis at 22 weeks of gestation and was treated with antibiotics. Her breathing difficulties and stridor, present during both inspiration and expiration, progressively worsened. She was referred to the Departments of Thoracic Surgery and Otorhinolaryngology at B University Hospital, where a stent or tracheostomy was proposed as treatment options.

The patient had no history of tracheal intubation or tracheostomy.

At 28 weeks and five days of gestation, she consulted the Departments of Obstetrics and Otorhinolaryngology - Head and Neck Surgery at our university hospital, as continuous respiratory monitoring at Obstetrics Clinic A was deemed challenging.

Clinical findings and initial assessment

On initial examination, she exhibited respiratory distress, characterized by difficulty breathing during both inspiration and expiration, stridor, and dyspnea on exertion (DOE). Her vital signs included a respiratory rate of 18 breaths per minute, a heart rate of 86 beats per minute, a blood pressure of 111/67 mmHg, and a peripheral oxygen saturation of 98% on room air. Breath sounds were audible in the neck, and her anterior neck was visibly sunken during inspiration.

Flexible Endoscopic Findings at the First Visit

**Figure 1:**
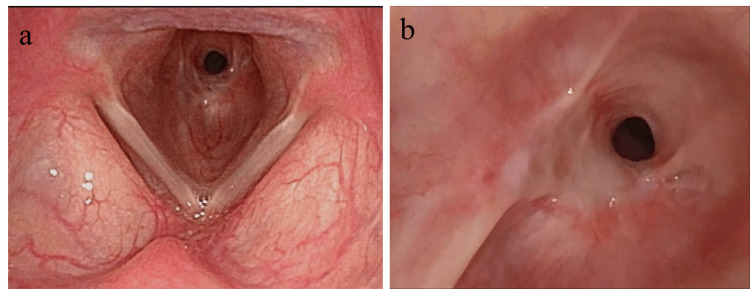
Endoscopic images from the initial visit (a) Circumferential stenosis of the subglottic region at the level of the cricoid cartilage. (b) A close-up view showing circumferential and cicatricial stenosis of the larynx.

Flexible endoscopy revealed circumferential stenosis of the subglottic larynx at the level of the cricoid cartilage (Figure 1a, 1b).

Diagnostic workup

CT Findings

**Figure 2:**
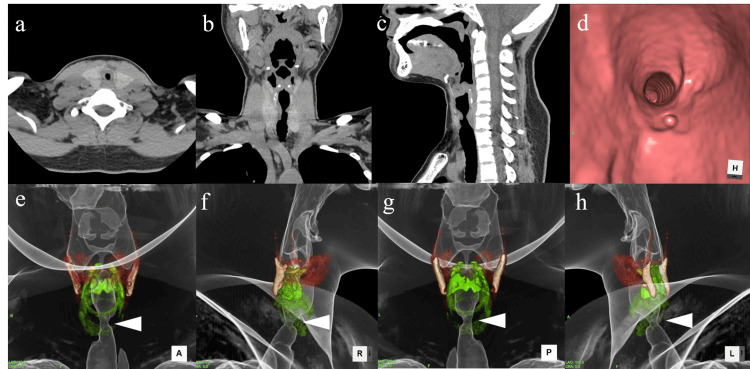
CT findings (a) Axial image. (b) Coronal image. (c) Sagittal image. (d) Virtual endoscopic image. 3D reconstruction images: (e) Anterior view. (f) Right-side view. (g) Posterior view. (h) Left-side view. The subglottic area was narrowed.

**Figure 3:**
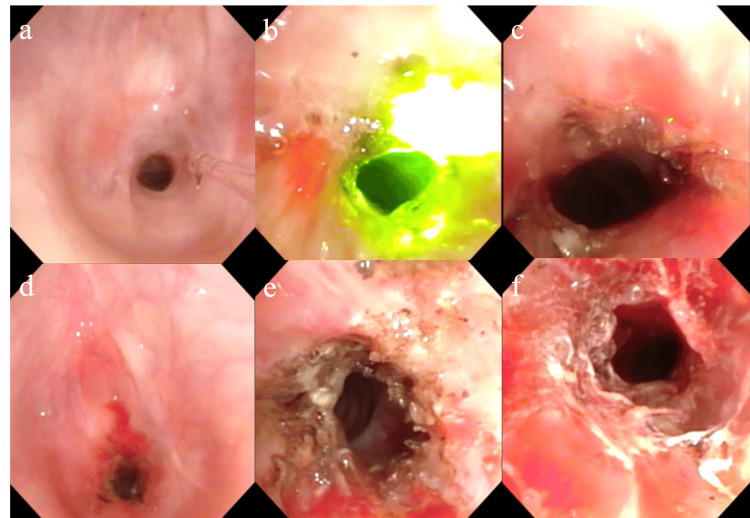
Intraoperative findings (a) Before laser treatment. (b) During green light laser application. (c) Close-up view. (d) Distant view. (e) After green light laser ablation. (f) After triamcinolone acetonide injection.

CT imaging was performed to assess the extent of tracheal narrowing and determine the feasibility of endoscopic laser ablation. After consulting an obstetrician, we proceeded with the CT scan, minimizing the scan area and protecting the abdomen to reduce fetal radiation exposure. The airway was narrowed to approximately 5 mm in diameter, with stenosis affecting the anterior left side from the first to the fourth tracheal rings and the right side from the first to the second rings (Figure 2a, 2b, 2c). Virtual endoscopic images (Figure 2d) and 3D reconstructed imaging (Figure 2a, 2b, 2c, 2d) were obtained. No tracheal cartilage thickening was observed.

Differential Diagnosis

Autoimmune diseases, such as granulomatosis with polyangiitis (formerly Wegener’s granulomatosis) and relapsing polychondritis (RP), were considered. Since ISGS is a diagnosis of exclusion, she underwent blood tests and an auricular cartilage biopsy. RP is often diagnosed early through an auricular biopsy. Laboratory tests revealed only mild anemia and an elevated inflammatory response (Table 1). A biopsy of the left auricular cartilage at 31 weeks and three days showed no inflammatory changes, ruling out RP.

**Table 1:** Laboratory data Alb: albumin; ANA: antinuclear antibody; β2-MG: beta2-microglobulin; BUN: blood urea nitrogen; C3: complement 3; C4: complement 4; Cr: creatinine; FT3: free thyroxine 3; FT4: free thyroxine 4; Hb: hemoglobin; HbA1c: hemoglobin A1c; Ht: hematocrit; MPO-ANCA: myeloperoxidase-anti-neutrophil cytoplasmic antibodies; NAG: N-acetyl-β-D glucosaminidase; Plt: platelet; PR3-ANCA: proteinase-3-anti-neutrophil cytoplasmic antibodies; RF: rheumatoid factor; TSH: thyroid-stimulating hormone; TP: total protein

Parameter	Value	Unit
Hematology		
WBC	5.9 × 10³	/μL
RBC	3.36 × 10⁶	/μL
Ht	31.1	%
Hb	10.5	g/dL
Plt	26.8 × 10⁴	/μL
Electrolytes and renal function		
Na	137	mmol/L
K	4	mmol/L
Cl	103	mmol/L
BUN	5	mg/dL
Cr	0.45	mg/dL
Protein and immunology		
TP	6.9	g/dL
Alb	3.3	g/dL
IgG4	32	U/L
CRP	0.07	mg/dL
Complement C3	136	mg/dL
Complement C4	18	mg/dL
IgG	1,332	mg/dL
IgA	231	mg/dL
IgM	51	mg/dL
Urinalysis		
Protein	(-)	
Blood	(-)	
Sugar	(-)	
β2-m	217	μg/L
NAG	2.6	U/L

Diagnosis

ISGS was diagnosed based on clinical, endoscopic, and histopathological findings, after ruling out other possible causes.

Treatment and management

At 31 weeks and three days, a multidisciplinary team - including obstetricians, anesthesiologists, endocrinologists, and otolaryngologists - discussed the treatment plan. However, the patient declined surgical intervention, including tracheostomy, and opted to start oral prednisolone despite negative results for polyangiitis and other autoimmune conditions. Since her respiratory symptoms and flexible endoscopic findings did not improve, we proceeded with laser ablation and local steroid injection for SGS under local anesthesia at 33 weeks and five days.

Surgical treatment

Obstetricians conducted fetal monitoring, while anesthesiologists managed respiration and pain control. A flexible laryngeal endoscope was inserted through the nose with the patient in a supine position. Local anesthesia with 4.0% lidocaine was administered to the pharynx, larynx, and subglottic stenotic area. SGS was visualized and treated with laser ablation using a green laser (Lumenis Be Ltd., Yokneam Illit, Israel). The laser was set to 2.3W in pulse mode and applied 1,786 times.

Laser ablation began from the 0 to 3 o’clock position (anterior to the right side of the trachea) and continued from the 7 to 12 o’clock position (left to posterior side). After ablation, excess tissue was removed using flexible laryngeal endoscopic forceps. Finally, 1.0 mL of triamcinolone acetonide was injected into the anterior (0°), right (3°), and left (9°) positions. The patient’s respiratory symptoms improved, and the procedure was completed successfully (Figure 3a, 3b, 3c, 3d). The total operative time was one hour and 26 minutes.

Histopathological findings

Histological examination of the subglottic laryngeal biopsy tissue revealed fibrosis and mild inflammatory changes. The mucosa was covered with pseudostratified columnar epithelium, with fibrosis and fibroblast proliferation beneath the epithelium. There were no signs of vasculitis, amyloid deposits, or IgG4-positive cells.

Clinical course after surgery

**Figure 4:**
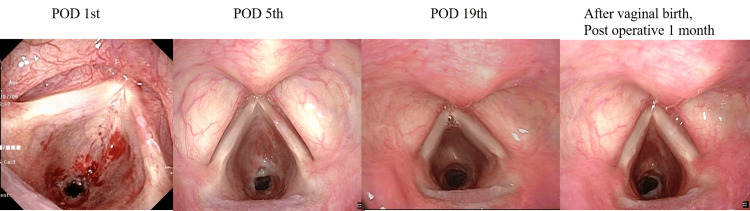
Postoperative endoscopic findings (a) Postoperative day 1. (b) Postoperative day 5. (c) Postoperative day 19: the SGS gradually widened. (d) One month after vaginal birth: the SGS area was wider than the preoperative view. SGS: subglottic stenosis

The patient was discharged without complications on the third postoperative day. At three weeks postoperatively (36 weeks of gestation), stridor was no longer audible, and DOE had resolved (Figure 4a, 4b, 4c). She had a spontaneous vaginal delivery at 38 weeks and three days, giving birth to a healthy boy (Figure 4d).

## Discussion

This case report describes the clinical features, flexible endoscopic imaging findings, and treatment of ISGS during pregnancy. Endoscopic treatment is both essential and safe for pregnant women and their fetuses.

Causes of SGS

SGS is a circumferential narrowing of the subglottic larynx and proximal trachea. It is more common in middle-aged women [7] and has been reported in pregnant women as well. While tracheal intubation, Wegener’s granulomatosis (polyangiitis), RP, and endotracheal tumors have been identified as causes of SGS in pregnancy, ISGS of unknown origin is extremely rare. To date, only 36 cases (across 16 studies) have been reported in English-language literature [5-19]. Notably, most ISGS cases in pregnant women have been documented in the UK, the USA, and Canada (12 out of 16 studies), with the majority involving Caucasian women. Tapias et al. also noted a predominance of ISGS in Caucasian women [6]. In contrast, only four cases have been reported in Asia, specifically in India and Iran [11-14], suggesting potential racial differences in its prevalence. To our knowledge, this is the first report of ISGS in Japan.

Inflammation and scarring are commonly observed in ISGS, making it essential to distinguish it from stenosis associated with granulomatosis with polyangiitis. In our case, laboratory tests showed mild inflammation and anemia, while histopathological examination of auricular cartilage and tracheal tissue revealed no signs of vasculitis. Based on these findings, the patient was diagnosed with ISGS during pregnancy.

Symptoms

Most patients with ISGS present with airway symptoms such as stridor, shortness of breath, and DOE. Additionally, cough is reported in nearly half of cases, with some patients experiencing wheezing, hoarseness, and breathing difficulties resembling asthma. There have been instances where patients were initially treated for asthma before an SGS diagnosis was established. In our case, the patient was diagnosed with asthma during her first pregnancy. After childbirth, her respiratory symptoms improved, and she was monitored without intervention. However, during her second pregnancy, her symptoms recurred and progressively worsened.

Pregnancy itself can exacerbate respiratory conditions due to diaphragm elevation, increased secretions, and tracheal mucosal edema. Hypoxemia can further impact fetal health, potentially leading to complications such as placental abruption, preeclampsia, and intrauterine growth restriction [12,16]. Given these risks, determining the optimal timing for treatment should be based on the severity of symptoms and other clinical findings.

Radiographic findings

Laryngeal endoscopy remains the gold standard for diagnosing SGS, as it provides direct visualization of subglottic narrowing [2]. The stenotic segment in ISGS typically measures between 1 and 3 cm in thickness, with a median length of 1.75 cm [6]. In our case, CT imaging revealed that the left anterior portion of the stenotic region was approximately 5 mm thick. Virtual endoscopic images, reconstructed from CT scans, closely resembled intraoperative findings (Figure 3d, Figure 4a). CT imaging can offer additional insights into stenotic thickness when compared to laryngeal endoscopy, and its utility should be considered based on the gestational age.

Treatment

While some pregnant women with ISGS have successfully delivered without intervention, most undergo endoscopic surgery under local anesthesia. Fang and Pai recommend endoscopic treatment as the first-line approach before considering open surgical procedures [7]. Among reported cases, 22 out of 33 patients (66.6%) underwent balloon dilation, while seven patients (21.2%) received multiple treatments, including laser therapy, mitomycin C injection, stent placement, and tracheal resection. Two patients required tracheostomy or laryngeal mask placement until delivery [5-19].

Treatment during pregnancy carries risks for both mother and fetus, including increased rates of preterm delivery, intrauterine growth restriction, and low birth weight [5]. Thus, surgical management must be carefully evaluated. In previous cases, laser therapy was used before balloon dilation. In our case, complete circumferential stenosis was treated with laser ablation, followed by a steroid injection. We employed a green contact-type laser, which enabled safe ablation under local anesthesia, even in the mobile tracheal region affected by respiration [20]. However, laser ablation has some limitations, including a longer operative time compared to balloon dilation and a delayed onset of therapeutic effects. The effects of the local steroid injection were observed to improve gradually.

In this patient, symptoms resolved approximately three weeks after surgery. By the time of delivery (33 days post-surgery), the SGS had further improved, and the baby was delivered spontaneously without complications. While respiratory symptoms related to pregnancy may subside after childbirth, treating SGS in women of childbearing age before pregnancy appears to be a safer approach than intervention during pregnancy.

## Conclusions

ISGS during pregnancy is extremely rare, and treatment strategies are based on limited case reports. Therapeutic endoscopic management, particularly balloon catheter dilation, is the most commonly used approach in pregnant women. In this case, a pregnant woman underwent endoscopic laser ablation combined with localized steroid injection for SGS. The stenosis gradually improved, and she was able to deliver spontaneously.

When treating pregnant women, it is crucial to consider potential effects on the fetus. The timing and method of treatment should be carefully planned through a multidisciplinary approach involving obstetrics, anesthesiology, and otorhinolaryngology.
